# Pathophysiological Links Between Inflammatory Bowel Disease and Cardiovascular Disease: The Role of Dysbiosis and Emerging Biomarkers

**DOI:** 10.3390/biomedicines13081864

**Published:** 2025-07-31

**Authors:** Roko Šantić, Nikola Pavlović, Marko Kumrić, Marino Vilović, Joško Božić

**Affiliations:** 1 Department of Pathophysiology, University of Split School of Medicine, 21000 Split, Croatia; roko.santic@mefst.hr (R.Š.); nikola.pavlovic@mefst.hr (N.P.); marko.kumric@mefst.hr (M.K.); josko.bozic@mefst.hr (J.B.); 2 Laboratory for Cardiometabolic Research, University of Split School of Medicine, Soltanska 2A, 21000 Split, Croatia

**Keywords:** cardiovascular diseases, inflammatory bowel disease, gut microbiota, biomarkers

## Abstract

This review introduces a novel integrative framework linking gut dysbiosis, systemic inflammation, and cardiovascular risk in patients with inflammatory bowel disease (IBD). We highlight emerging biomarkers, including short-chain fatty acids (SCFAs), calprotectin, and zonulin, that reflect alterations in the gut microbiome and increased intestinal permeability, which contribute to cardiovascular pathology. Cardiovascular diseases (CVDs) remain the leading cause of morbidity and mortality worldwide, and recent evidence identifies IBD, encompassing ulcerative colitis (UC) and Crohn’s disease (CD), as a significant non-traditional risk factor for CVD. This review synthesizes current knowledge on how dysbiosis-driven inflammation in IBD patients exacerbates endothelial dysfunction, hypercoagulability, and atherosclerosis, even in the absence of traditional risk factors. Additionally, we discuss how commonly used IBD therapies may modulate cardiovascular risk. Understanding these multifactorial mechanisms and validating reliable biomarkers are essential for improving cardiovascular risk stratification and guiding targeted prevention strategies in this vulnerable population.

## 1. Introduction

Cardiovascular diseases (CVDs) encompass a broad group of disorders affecting the heart and blood vessels [1,2]. They represent the leading cause of morbidity and mortality among adults worldwide, with a continually rising incidence [1]. Coronary artery disease and stroke are the most prominent contributors to cardiovascular-related deaths [1]. Well-established risk factors for CVD include advanced age, smoking, diabetes mellitus, obesity, hypertension, and hyperlipidemia. Cardiovascular risk factors can be broadly categorized as non-modifiable (such as age, sex, and genetic predisposition) and modifiable (including smoking, diet, physical inactivity, obesity, hypertension, and diabetes mellitus). While non-modifiable factors set the baseline risk, modifiable factors are particularly important in IBD patients, as chronic inflammation may amplify their adverse effects on the vasculature. For example, smoking not only exacerbates IBD but also independently increases cardiovascular risk. Similarly, physical inactivity and poor dietary habits can contribute to both gut dysbiosis and metabolic syndrome, further compounding CVD risk in this population [2,3]. In addition to these traditional risk factors, growing evidence suggests a range of novel contributors to cardiovascular risk [4].

Among these emerging risk factors, chronic kidney disease, non-alcoholic fatty liver disease, and inflammatory bowel disease (IBD) have been identified as essential conditions associated with increased cardiovascular morbidity. These associations are believed to be mediated, at least in part, by chronic systemic inflammation and alterations in gut microbiota composition, underscoring the importance of non-traditional mechanisms in the pathogenesis of cardiovascular disease [5,6,7,8,9,10].

IBD is a group of chronic inflammatory disorders that affect the gastrointestinal tract, characterized by recurrent episodes of inflammation. IBD encompasses two distinct clinical entities—UC and CD, each with its own specific clinical, histological, and molecular features [11,12,13,14,15]. In recent years, the global incidence of IBD has been steadily increasing, with the highest rates observed in Europe, ranging from 10.5 to 46.14 cases per 100,000 individuals annually [15,16]. Due to the high costs associated with long-term treatment and management, IBD has become a significant burden on healthcare systems [17]. The precise etiology of IBD remains incompletely understood. Still, current evidence suggests a multifactorial origin involving complex interactions among genetic predisposition, gut microbiota composition, environmental influences, and dysregulated immune responses [18,19]. While IBD primarily presents with gastrointestinal symptoms such as diarrhea and abdominal pain, extraintestinal manifestations are observed in up to 50% of patients. These commonly affect the musculoskeletal system (e.g., arthritis, enthesitis) and the skin (e.g., erythema nodosum, psoriasis, pyoderma gangrenosum), but may also involve other organs, including the kidneys, eyes, and cardiovascular system [20,21,22,23,24,25,26,27,28,29,30,31,32,33,34,35,36,37,38,39,40,41,42,43,44,45,46,47,48,49,50,51,52,53,54,55,56,57,58,59,60,61,62,63,64,65,66,67,68,69,70,71,72,73,74,75,76,77,78,79,80,81,82,83,84,85,86,87,88,89,90,91,92,93,94,95,96,97,98,99,100,101,102,103,104,105,106,107,108,109,110,111,112,113,114,115,116,117,118,119,120,121,122]. Central to both the pathogenesis of IBD and its systemic effects is the gut microbiome, a dynamic community of billions of microorganisms inhabiting the human gastrointestinal tract [23,24]. Far from static, the composition of the gut microbiota is shaped by factors such as diet, antibiotic use, and aging, and it undergoes continuous changes throughout life [24]. The gut microbiota plays a crucial role in regulating various physiological processes, including the metabolism of bile acids and short-chain fatty acids, maintaining intestinal barrier integrity, and modulating immune and inflammatory responses [25,26,27,28]. These functions highlight their essential contribution to overall human health. The use of pH 7.4 in experimental and clinical studies reflects the near-neutral environment of the distal colon, which is relevant for evaluating the stability and activity of gut-derived biomarkers and therapeutic agents in IBD [29].

**Figure 1:**
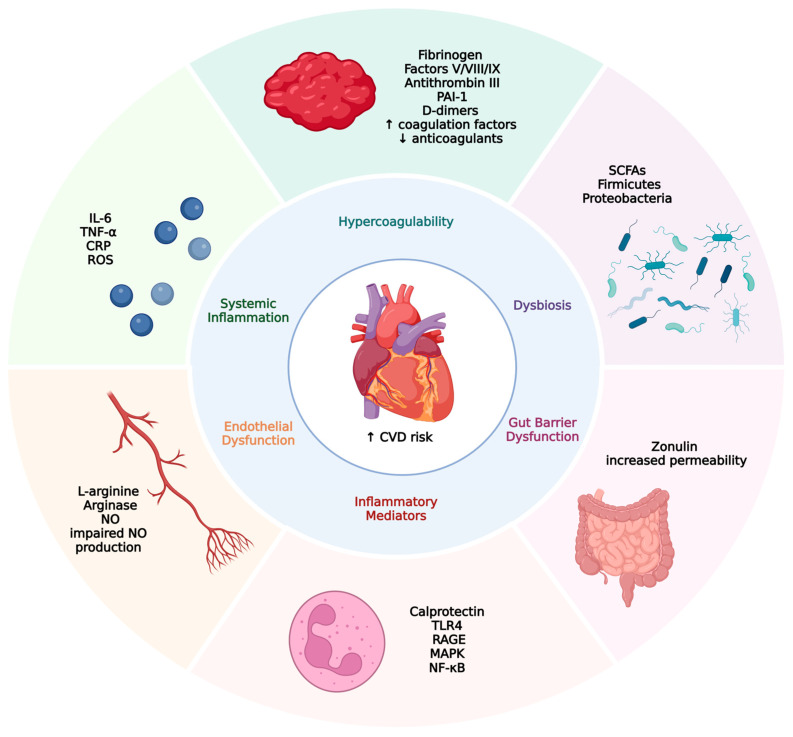
Pathophysiological links between inflammatory bowel disease and increased cardiovascular risk. This figure illustrates the interconnected mechanisms by which gut dysbiosis and gut barrier dysfunction contribute to an increased risk of cardiovascular disease (CVD). Gut dysbiosis, characterized by a reduction in short-chain fatty acid (SCFA)-producing bacteria and an increase in proinflammatory taxa, disrupts intestinal homeostasis. Zonulin-mediated increased gut permeability enables the translocation of endotoxins into the systemic circulation, triggering systemic inflammation. Elevated levels of proinflammatory cytokines (e.g., IL-6, TNF-α) and reactive oxygen species (ROS) promote endothelial dysfunction and activate inflammatory pathways (e.g., TLR4, NF-κB). Concurrently, hypercoagulability develops due to elevated coagulation factors and reduced natural anticoagulants. These interconnected processes culminate in increased cardiovascular risk, including thrombosis, atherosclerosis, vascular stiffness, and cardiac remodeling.

The gut microbiota is primarily composed of five dominant bacterial phyla: *Actinobacteria*, *Bacteroidetes*, *Verrucomicrobia*, *Firmicutes*, and *Proteobacteria*, with *Bacteroidetes* and *Firmicutes* together accounting for up to 90% of the total gut bacterial population [29]. Alterations in the diversity and abundance of these microbial communities, a state known as dysbiosis, have been positively associated with systemic inflammation, insulin resistance, and increased intestinal permeability [25,30]. As a result, dysbiosis has become a key area of interest in the pathophysiology of numerous chronic conditions, particularly inflammatory bowel disease, type 2 diabetes mellitus, and chronic kidney disease [31,32]. In contrast to dysbiosis, the term eubiosis refers to a balanced and diverse gut microbiota composition that supports intestinal homeostasis, effective immune regulation, and metabolic health. Eubiosis is characterized by the predominance of beneficial microbial taxa, optimal production of short-chain fatty acids, and maintenance of mucosal barrier integrity, all of which are essential for preventing chronic inflammation and related systemic diseases, including IBD and cardiovascular disease [33,34]. The key pathophysiological mechanisms linking gut dysbiosis and increased cardiovascular risk in IBD patients are illustrated in Figure 1.

To understand the full spectrum of cardiovascular risk in IBD patients, it is essential to examine both shared pathophysiological mechanisms and emerging gut-related biomarkers. Systemic inflammation represents a critical communication axis between the gut microbiota and host, mediating the increased cardiovascular risk observed in IBD. Recent advances in predictive and personalized medicine highlight the significance of the gut–vascular axis in the development of cardiovascular complications among IBD patients [35].

We hypothesize that gut dysbiosis is a central mediator, among several parallel pathways, linking IBD and increased cardiovascular risk. This review advances a model in which alterations in microbiota composition drive systemic inflammation, endothelial dysfunction, and hypercoagulability, with host- and microbe-derived biomarkers serving as both indicators and mediators of disease progression. Emerging evidence suggests that the increased cardiovascular risk observed in patients with inflammatory bowel disease (IBD) results from a complex interplay between genetic susceptibility, environmental exposures, and alterations in the gut microbiota. Genome-wide association studies have identified multiple genetic loci that confer increased risk for both IBD and cardiovascular disease, implicating shared inflammatory and immune pathways in their pathogenesis. Environmental factors, such as dietary patterns, antibiotic use, and lifestyle choices, are known to shape the composition and function of the gut microbiota, leading to dysbiosis—a state characterized by reduced microbial diversity and an imbalance between beneficial and pathogenic bacteria. Dysbiosis in IBD patients promotes increased intestinal permeability, facilitating the translocation of microbial products into circulation and thereby amplifying systemic inflammation. This contributes to endothelial dysfunction, hypercoagulability, and atherosclerosis [36,37]. Given these interconnections, this review aims to explore the impact of inflammatory bowel disease on the cardiovascular system, with a particular focus on identifying biomarkers associated with gut dysbiosis that may mediate or contribute to cardiovascular pathology. While dysbiosis is recognized as a relevant pathophysiological mechanism, this review will focus on specific microbial or host-derived markers that may influence cardiovascular risk through the mechanism of dysbiosis.

## 2. Pathophysiological Links Between IBD and CVD

### 2.1. Hypercoagulability and Thrombosis Risk

Hypercoagulability is a well-established risk factor associated with the development of venous, arterial, and microvascular thrombosis [38]. Although up to 30% of patients with venous thromboembolism (VTE) have inherited mutations, the majority of cases are still considered to be acquired [39,40]. Patients with inflammatory bowel disease (IBD) are regarded as being at increased risk for VTE, with numerous studies reporting a higher incidence ranging from 3.8% to 8%. Some studies have shown an even higher incidence in patients with Crohn’s disease and active disease [41,42].

Several alterations in blood coagulation may explain this phenomenon. Firstly, patients with IBD have been shown to exhibit elevated levels of various coagulation factors, including fibrinogen, factors V, VIII, and IX, in addition to a deficiency in natural anticoagulants such as antithrombin III [41,43,44,45].

Secondly, hypofibrinolysis, a condition characterized by the impaired ability to degrade fibrin clots, also contributes to the prothrombotic state [46]. Fibrinolysis is primarily mediated by the serine protease plasmin, which must first be activated from its precursor plasminogen [47]. Several proteins tightly regulate this activation process. The conversion of plasminogen to plasmin is positively regulated by tissue-type and urokinase-type plasminogen activators, while it is negatively regulated by plasminogen activator inhibitors 1 and 2 (PAI-1 and PAI-2) [48,49]. Studies have shown increased levels of PAI-1, and some have even reported the presence of antibodies against tissue plasminogen activator (t-PA). Elevated plasminogen levels have also been observed, possibly due to increased PAI-1 levels that inhibit its activation, as well as the fact that plasminogen is an acute-phase protein [50,51,52]. Additionally, elevated levels of D-dimers have been observed [43,53]. D-dimers are fibrin degradation products that are clinically used to exclude VTE; however, their utility extends beyond diagnosis. They are also predictive of long-term risk for both arterial and venous thrombotic events and cardiovascular disease (CVD) mortality [54].

Given the heightened risk of thromboembolic complications in IBD, current clinical guidelines recommend the use of pharmacological thromboprophylaxis in hospitalized patients with moderate to severe disease activity, especially during flares or periods of immobilization [55,56]. However, the optimal duration and intensity of prophylaxis in the outpatient setting remain as subjects of ongoing research. While systemic inflammation is a major driver of hypercoagulability in IBD, emerging evidence suggests that specific microbiota-derived mediators, such as trimethylamine N-oxide (TMAO), may independently promote thrombosis. Recent meta-analyses have begun to dissect these pathways, indicating that both inflammatory and microbial factors contribute to the heightened thrombotic risk in IBD [57]. Recent research has highlighted that hypercoagulability in IBD arises from both systemic inflammation and the direct effects of microbiota-derived mediators, with each contributing through distinct yet interconnected pathways. Systemic inflammation, characterized by elevated circulating cytokines such as IL-6, TNF-α, and CRP, upregulates the hepatic synthesis of procoagulant factors (e.g., fibrinogen, factor VIII) while suppressing natural anticoagulants, including antithrombin III, thereby creating a prothrombotic milieu [58,59]. This inflammatory state also induces endothelial dysfunction, further promoting platelet activation and aggregation. In parallel, gut dysbiosis and increased intestinal permeability facilitate the translocation of bacterial products, such as lipopolysaccharide (LPS), into the systemic circulation. LPS and other microbial metabolites can directly activate the coagulation cascade via toll-like receptor 4 (TLR4) signaling on endothelial cells and platelets, independently of traditional inflammatory cytokines. Recent studies have shown that LPS exposure increases tissue factor expression and enhances thrombin generation, providing a mechanistic link between microbial dysbiosis and hypercoagulability [60].

In summary, the prothrombotic state observed in IBD results from a complex interplay among inflammatory, coagulation, and fibrinolytic pathways, and is associated with a significantly increased risk of both venous and arterial thrombotic events. Further research is needed to delineate better the mechanisms involved and to develop targeted strategies for risk stratification and prevention in this vulnerable patient population.

### 2.2. Chronic Inflammation and Atherosclerosis

Atherosclerosis is a progressive vascular disease characterized by fibrofatty lesions within the arterial wall, representing the underlying cause of cardiovascular diseases. The process of atherogenesis originates with endothelial activation, which facilitates the accumulation of lipids, calcium, and inflammatory cells [61,62]. Inflammatory bowel disease (IBD) is increasingly recognized as an essential risk factor for the accelerated development of atherosclerosis, similar to other chronic inflammatory diseases such as rheumatoid arthritis, systemic lupus erythematosus, and systemic sclerosis [63,64]. The underlying pathophysiology is complex and includes several traditional risk factors such as obesity, hypertension, type 2 diabetes mellitus, and hypercholesterolemia [62,65]. However, these factors alone are insufficient to fully explain the mechanisms involved, as significant atherosclerosis has also been observed in low-risk IBD patients [66]. Sleutjes et al. reported higher odds of hypertension (OR 1.67, 95% CI 1.19–2.32), increased waist circumference (+4 cm, *p* = 0.006), and elevated triglyceride levels (+0.6 mmol/L, *p* < 0.001) in IBD patients compared to healthy controls [67]. Regarding type 2 diabetes, Jesse et al. demonstrated a higher risk in patients with IBD (SIR 1.54; 95% CI 1.49–1.60) [68]. Similarly, a study by Kang et al. found an increased risk of diabetes in patients with Crohn’s disease (HR 1.677; 95% CI 1.408–1.997), with the effect being more pronounced in younger individuals [69].

Hypercholesterolemia, particularly elevated levels of low-density lipoprotein (LDL) cholesterol, is considered a significant risk factor in the progression of atherosclerosis [62,70]. However, in IBD patients, as well as in other chronic inflammatory conditions such as rheumatoid arthritis, the “lipid paradox” has been observed [71,72,73]. This phenomenon describes an inverse correlation between lipid values and disease activity. A 2023 meta-analysis demonstrated lower levels of total cholesterol (TC), high-density lipoprotein (HDL), and LDL in IBD patients compared to controls. Additionally, lower levels of TC and LDL were reported in patients with active IBD and non-mild UC compared to those with inactive IBD and mild UC [71]. These findings should be interpreted with caution, as an extensive cohort study demonstrated an inverse correlation between all-cause mortality and LDL levels in patients with high inflammatory risk defined by hs-C-reactive protein (CRP) > 3 mg/L [74]. The mechanism behind this paradox is not yet fully understood, although current data suggest a role for inflammation-driven hypercatabolic states of LDL [75].

The most significant impact on atherogenesis arises from chronic systemic inflammation, mediated through multiple mechanisms including increased oxidative stress, elevated levels of circulating cytokines, and activation of the innate immune system [76,77,78]. Oxidative stress is a hallmark of IBD, characterized by elevated levels of reactive oxygen species (ROS), which exert harmful effects [79,80]. Damage to intracellular components—including DNA, the endoplasmic reticulum, cell membranes, and mitochondria—can all be attributed to oxidative stress [79,81]. ROS contribute to atherogenesis at multiple levels [82]. Oxidized LDL (oxLDL) promotes atherosclerosis via several mechanisms, including the transformation of macrophages into foam cells through scavenger receptors and the activation of vascular smooth muscle cells, resulting in their proliferation and migration [83]. Excess reactive oxygen species (ROS) are associated with impaired plaque stability, explained by upregulation of matrix metalloproteinases that degrade collagen within the plaque, as well as ROS-induced macrophage apoptosis [82,84].

Proinflammatory cytokines represent another key mediator of inflammation, contributing to all stages of atherogenesis [77]. Cytokines such as IL-1, IL-6, and TNF-α, shown to be elevated in IBD patients, exert multiple effects including endothelial activation, altered permeability, immune cell adhesion and proliferation, degradation of the fibrous cap, and increased plaque instability [85,86,87].

Another necessary consequence of systemic inflammation is endothelial dysfunction, which refers to the impairment of numerous endothelial functions, including metabolism, angiogenesis, and regulation of vascular tone [88]. In clinical practice, macrovascular endothelial function is most commonly assessed using the flow-mediated dilation (FMD) test, despite its relatively low sensitivity. FMD measures the increase in blood flow in the brachial artery resulting from elevated shear stress following exposure to suprasystolic pressure. This post-ischemic vasodilation is mediated by nitric oxide (NO) production, which reflects endothelial functionality [89]. Using the FMD test, numerous studies have demonstrated impaired endothelial function in IBD patients, with no significant difference observed CD and UC [90,91,92]. The pathophysiology involved in this mechanism is complex, with evidence suggesting a central role of systemic inflammation. This is supported by findings that endothelial dysfunction positively correlates with CRP levels [93,94]. IBD patients exhibit increased activity of arginase, an enzyme that competes with endothelial nitric oxide synthase (eNOS) for the common substrate L-arginine, thereby reducing the bioavailability of L-arginine for NO production [95,96,97]. Moreover, proinflammatory cytokines such as tumor necrosis factor (TNF) induce the expression of arginase [98,99]. The importance of NO extends beyond regulating vascular tone, as it also exerts beneficial anti-atherogenic effects, including the inhibition of platelet aggregation and leukocyte adhesion [100,101]. Given its involvement in early vascular changes and its correlation with systemic inflammatory markers, endothelial dysfunction represents a valuable early indicator and surrogate marker of atherosclerosis [102,103]. These mechanistic insights are summarized in Table 1, which outlines the key pathophysiological pathways by which inflammatory bowel disease contributes to cardiovascular risk.

## 3. Impact of IBD Medications on Cardiovascular Health

The treatment of inflammatory bowel disease (IBD) relies heavily on the lifelong use of various medications that suppress and modulate inflammation. The primary aim of these drugs is to induce and maintain remission. The most commonly used agents include corticosteroids, aminosalicylates, azathioprine, and biological agents such as TNF-alpha antagonists [117,118,119,120]. Although the benefits of therapy generally outweigh the adverse effects, side effects are still common. Various studies report that approximately 50% of patients experience at least one adverse event during biological therapy, most commonly mild administration-related reactions, followed by skin and subcutaneous tissue disorders [121]. Godat et al. reported that 67.8% of 3138 patients experienced one or more drug-related side effects requiring treatment discontinuation [122].

Among these therapies, corticosteroids, primarily used to induce remission, have well-established adverse effects [112]. Corticosteroids can cause dyslipidemia, characterized by elevated total cholesterol, LDL, and triglyceride levels, by promoting insulin resistance and impairing lipid metabolism in the liver [123,124,125]. Additionally, by fostering fluid retention and increasing total peripheral resistance, hypertension is another documented adverse effect [126]. One study showed that even low oral doses of corticosteroids are associated with significantly increased hazard ratios: 1.69 (95% CI 1.54–1.85) for atrial fibrillation, 1.75 (95% CI 1.56–1.97) for heart failure, 1.76 (95% CI 1.51–2.05) for acute myocardial infarction, 1.78 (95% CI 1.53–2.07) for peripheral arterial disease, 1.32 (95% CI 1.15–1.50) for cerebrovascular disease, and 1.93 (95% CI 1.47–2.53) for abdominal aortic aneurysm [126]. Corticosteroids remain widely used in IBD treatment, with 41% of patients with UC and 57% with CD receiving them within five years of diagnosis. Notably, up to 25% of patients experience prolonged use, and nearly 50% of prescriptions may be avoidable [127].

5-Aminosalicylic acid (5-ASA) is another frequently used drug in the treatment of IBD, recognized for its favorable safety profile [128,129]. Nevertheless, cases of drug-induced myocarditis and pericarditis have been reported, most commonly associated with mesalamine and other 5-ASA derivatives [130,131]. In contrast to corticosteroids, a Danish cohort study reported a lower risk of ischemic heart disease in 5-ASA users compared to non-users [132]. While some studies suggest a protective cardiovascular effect of 5-ASA, others report neutral or even adverse outcomes, particularly in patients with extensive disease or comorbidities. The impact of biologics on cardiovascular risk is highly context-dependent, varying with treatment duration, patient phenotype, and underlying inflammation [3,133].

Azathioprine is also a critical medication, frequently used to maintain remission, first-line in CD and second-line after 5-ASA in UC [134]. Schuchardt et al. demonstrated that prolonged azathioprine administration in rats led to vascular mineralization, implicating the NOD-, LRR- and pyrin domain-containing protein 3 (NLRP3) inflammasome pathway and associated inflammatory markers [135]. Conversely, Frostegård et al. concluded that azathioprine may exert protective anti-atherogenic effects by reducing T cell-driven vascular inflammation [134].

In step-up treatment strategies, biologics are generally reserved for patients whose disease remains dependent on steroid therapy [136]. The most commonly used agents include TNF inhibitors such as infliximab and adalimumab, and the integrin antagonist vedolizumab [137,138]. TNF-alpha exerts its effects through two types of receptors: TNFR1 and TNFR2. In the context of cardiovascular risk, TNFR1 stimulation is linked with pro-apoptotic and adverse inotropic effects, while TNFR2 stimulation is associated with cardioprotective effects and apoptosis prevention [139,140].

The ATTACH study, conducted in patients with heart failure, revealed increased mortality and hospitalization rates in those receiving high doses of infliximab (10 mg/kg) [141]. Although the recommended initial dose in IBD patients is 5 mg/kg, some require higher dosages [142]. Shehab et al., in a systematic review of 64 studies, found no clear association between the use of biologic therapies and major adverse cardiovascular events (MACEs); however, the lack of real-world data limits the ability to evaluate long-term risks fully [143].

In conclusion, IBD medications have well-established therapeutic benefits. Still, their use carries potential adverse effects on the cardiovascular system, which may be particularly serious given the emerging evidence suggesting that IBD itself contributes to increased cardiovascular risk. Continued evaluation through long-term, real-world data is essential to understand and balance these risks against the therapeutic benefits fully.

In recent years, increasing attention has been given to the role of the gut microbiome in shaping systemic inflammation and metabolic regulation. Dysbiosis in IBD patients not only exacerbates intestinal symptoms but may also influence cardiovascular outcomes through various microbial products and host responses. This section highlights three key biomarkers, short-chain fatty acids (SCFAs), calprotectin, and zonulin, that represent potential mechanistic links between gut dysbiosis and cardiovascular disease in the context of IBD (Table 2).

### 3.1. Short-Chain Fatty Acids (SCFAs): Gut Metabolites with Systemic Impact

Short-chain fatty acids (SCFAs), produced by beneficial gut bacteria, play a crucial role in maintaining intestinal barrier integrity and modulating immune responses. In our conceptual model, the reduction in SCFA-producing bacteria in IBD patients represents a core feature of dysbiosis. This deficit leads to increased gut permeability, facilitating the systemic spread of proinflammatory mediators and thereby linking dysbiosis directly to the heightened cardiovascular risk observed in this population [145]. In IBD, gut dysbiosis is characterized by a significant decrease in the abundance of key SCFA-producing bacteria, including *Faecalibacterium prausnitzii*, *Roseburia* spp., and members of the *Ruminococcaceae* and *Lachnospiraceae* families. This microbial shift results in reduced colonic SCFA synthesis, particularly butyrate, which is crucial for epithelial barrier integrity and immune homeostasis. The loss of these beneficial taxa directly contributes to the lower SCFA levels observed in IBD patients, linking dysbiosis to impaired gut and cardiovascular health [145,146,147]. SCFAs are formed in the fermentation of fibers in food and some carbohydrates that are resistant to bacterial fermentation. They include acetate, propionate, and butyrate [23,131]. In the gut, they play an essential role as an energy substrate for epithelial cells and in maintaining immune homeostasis [148]. They are produced primarily by (*Ruminococcus*, *Akkermansia*, *Bifidobacterium*, *Lactobacillus*, *Succinivibrio*, *Roseburia*, *Clostridium*, and *Eubacterium*) and mainly in the proximal parts of the colon, especially the cecum [144,149]. It is widely accepted that plasma levels of SCFA are significantly reduced in patients with IBD compared to healthy control groups [150,151,152]. Few mechanisms explain reduced levels of SCFA in IBD patients. It is primarily associated with dysbiosis and depletion of *Ruminococcaceae* and *Lachnospiraceae*, which are representatives of the *Firmicutes phylum*, as well as *F. prausnitzii* [153]. They are producers of butyrate in the gut, which accounts for 20% of SCFA, and it is the most well-understood fatty acid in terms of its beneficial effects [154]. Other mechanisms include changes in luminal pH and oxygen concentrations, deficiency in growth factors such as iron, and increased colon motility, which decreases the time for microbial fermentation [149,155].

SCFAs have a positive impact on the gut by maintaining intestinal barrier integrity, reducing inflammation, promoting mucus production, regulating gut motility, and decreasing the incidence of colon cancer [156,157]. SCFA shows many beneficial effects on cardiovascular health, and its depletion could explain some of the pathophysiology behind cardiovascular diseases in patients with IBD [158,159]. SCFAs may lower blood pressure through vasodilation, though their antihypertensive effect varies ethnically [160]. African populations, being more salt-sensitive, have hypertension that is more dependent on volume. Lower levels of *Ruminococcaceae* have been associated with higher blood pressure, underscoring the role of gut microbiota. Interestingly, high fecal SCFA levels correlate with hypertension, likely due to a large unabsorbed fraction, suggesting inefficiencies in SCFA utilization [161]. By improving glucose regulation, lowering blood pressure, and controlling inflammation within the vessel wall, SCFAs contribute to the reduction in atherosclerosis. They also inhibit the proliferation of arterial smooth muscle cells, preventing vascular remodeling and plaque progression [162]. Short-chain fatty acids (SCFAs) have been shown to inhibit cardiac fibrosis, serve as an efficient energy source for failing hearts, and restore mitochondrial function. While SCFAs are generally considered beneficial due to their anti-inflammatory and barrier-protective effects, recent evidence indicates that butyric acid may also promote vascular calcification by regulating histone deacetylase and NF-κB pathways. This highlights the importance of a nuanced understanding of SCFA biology in cardiovascular disease [163].

A study conducted on diabetic patients at Cipto Mangunkusumo Hospital found that fecal SCFA excretion is associated with an increased risk of cardiovascular disease. Using gas chromatography–mass spectrometry, they analyzed fecal SCFA. A correlation was found between fecal short-chain fatty acids and a higher incidence of peripheral artery disease and random blood glucose levels [164].

Another study comparing heart failure patients classified as New York Heart Association (NYHA) Class III and IV with a healthy control group found a significant decrease in the phylum *Firmicutes* among those with severe congestive heart failure (CHF). Additionally, in these patients, the phylum Proteobacteria was the second most abundant, replacing Bacteroides. The study also reported a reduced abundance of SCFA-producing bacteria, particularly those from the genera *Ruminococcaceae* and *Lachnospiraceae* [165].

In summary, SCFAs play a crucial role in maintaining intestinal and cardiovascular health, with their depletion linked to the pathophysiology of inflammatory bowel disease and cardiovascular disorders. The consistent association between reduced SCFA-producing bacteria and disease states underscores the importance of modulating gut microbiota as a potential therapeutic strategy.

### 3.2. Calprotectin: Marker and Mediator of Inflammatory Risk

Calprotectin is a calcium- and zinc-binding protein primarily produced by neutrophils during inflammation, serving as a sensitive marker of intestinal inflammation. It plays essential roles both intracellularly and extracellularly: within cells, calprotectin mitigates leukocyte migration via cytoskeletal remodeling, transports arachidonic acid to inflammatory sites, and influences gene expression in inflammatory and malignant conditions. Extracellularly, through receptor-mediated mechanisms, it promotes neutrophil recruitment, cytokine expression, and cell proliferation, amplifying the inflammatory response. In the context of IBD, elevated fecal calprotectin not only signals ongoing mucosal inflammation but also, as posited in our integrative framework, reflects a heightened systemic inflammatory state. This systemic inflammation is a critical driver of endothelial dysfunction and hypercoagulability, thereby bridging the gap between local gut pathology and increased cardiovascular risk in IBD patients [166,167].

Calprotectin is a well-established biomarker of inflammation, widely used in clinical practice due to its high sensitivity but relatively low specificity [168]. It is routinely measured in fecal samples, where elevated levels serve as an indicator of intestinal inflammation, making it a valuable tool for screening, monitoring disease activity, and detecting relapses in conditions such as inflammatory bowel disease (IBD) [168,169]. While fecal calprotectin remains the standard for assessing gut inflammation, recent studies have explored the potential utility of serum and plasma calprotectin in IBD monitoring, yielding variable success [170,171]. Further research is needed to clarify its clinical applicability and optimize its role in disease management.

Studies suggest that levels of calprotectin may have predictive value in CVD, independent of gut inflammation. Prospective study PREVEND reported that calprotectin is an independent inflammatory marker that shows a log-linear association with cardiovascular disease risk, and although this association is attenuated after adjustment for hsCRP, it remains statistically significant [172]. A recent systematic review and meta-analysis comprising 20 studies with 3300 coronary artery disease (CAD) patients and 1230 controls confirmed these findings, reporting that patients with CAD had significantly higher calprotectin levels (SMD 0.81, 95% CI 0.32–1.30, *p*  <  0.01), particularly in the context of acute coronary syndromes, supporting its potential role as both a diagnostic and prognostic biomarker in cardiovascular disease [173].

Systemic inflammation is a well-established independent risk factor for the development of cardiovascular diseases; however, emerging evidence suggests that calprotectin may play an active role beyond serving as a mere marker of inflammation [70,71,72,73]. In patients with antiphospholipid syndrome, calprotectin enhances the production of negatively charged phospholipids, such as phosphatidylserine, which in turn promotes a procoagulant state [174,175]. Other mechanisms of calprotectin’s action on the heart include its interaction with TLR4 and RAGE receptors, leading to activation of NF-κB and MAPK signaling pathways [176]. In cardiac fibroblasts, this results in elevated production of cytokines, proteases, and extracellular matrix proteins. Moreover, calprotectin binding to RAGE receptors on cardiomyocytes impairs calcium flux, ultimately reducing cardiomyocyte contractility [177].

Existing evidence suggests that there is a correlation between dysbiosis and levels of calprotectin in both IBD and non-IBD patients. Heinzel et al. reported significant alterations in the gut microbiome among older individuals with elevated fecal calprotectin levels in a large cohort. These changes included a reduced abundance of SCFA-producing bacteria and an increased presence of genera such as *Veillonella* and *Haemophilus*, which have previously been associated with inflammatory IBD flares. In addition to microbiome dysbiosis, individuals in the high calprotectin group also exhibited a higher incidence of acute myocardial infarction [178].

Another study, conducted by Shaw et al., demonstrated a significant association between gut dysbiosis and fecal calprotectin levels, reporting that a one-unit increase in the dysbiosis index was associated with a 286 μg/g increase in fecal calprotectin [179]. Evidence suggests that an imbalance in the *Akkermansia*-to-*Enterobacteriaceae* ratio is related to elevated fecal calprotectin, highlighting the microbiome’s role in IBD and the development of cardiovascular disease (CVD) [180]. An association has also been observed between elevated calprotectin levels and the B2 enterotype, which is characterized by a relative abundance of *Bacteroides* species and reduced microbial cell density [181,182].

In conclusion, calprotectin is emerging as both a cardiovascular and dysbiosis marker, with elevated levels linked to increased cardiovascular disease risk and an imbalance in gut microbiota, characterized by a proinflammatory microbial profile and altered cytokine expression.

### 3.3. Zonulin: Intestinal Barrier Dysfunction and Cardiovascular Implications

Zonulin is an endogenous homolog of the *Vibrio cholerae Zonula occludens* toxin (Zot) and plays a crucial role in modulating intestinal permeability [183]. It is primarily produced in the liver but can also be secreted by the small intestine upon stimulation of the CXCR3 receptor, which is activated by gliadin [184]. Mechanistically, elevated zonulin increases intestinal permeability, allowing microbial products such as lipopolysaccharide (LPS) to translocate into circulation. This endotoxemia activates inflammatory pathways, including TLR4 and NF-κB signaling, which promote endothelial dysfunction and atherogenesis [138]. Research has demonstrated that CXCR3 expression is upregulated in celiac disease and inflammatory bowel diseases, suggesting a potential link between zonulin dysregulation and these pathological conditions [179,180,181]. Zonulin is derived from its precursor molecule, prehaptoglobin-2, and exerts its effects by stimulating epidermal growth factor receptor 2 (EGFR2) [184]. This activation leads to cytoskeletal reorganization and increased intestinal permeability [185]. Notably, zonulin is the only known physiological modulator of tight junction permeability, underscoring its significance in gut barrier function and disease pathophysiology [186]. Zonulin primarily forms when gliadin stimulates CXCR3 receptors, leading to the production of pre-haptoglobin 2, which is now recognized as zonulin. Among the three human haptoglobin phenotypes, only pre-haptoglobin 2 is linked to intestinal permeability regulation [187]. Studies indicate a higher prevalence of haptoglobin 2 and CXCR3 expression in patients with IBD compared to healthy individuals. Besides gliadin, bacterial stimulation can also increase zonulin levels by either upregulating CXCR3 expression or through direct interaction [146,188]. Calprotectin and zonulin differ in their specificity and predictive value for cardiovascular events. Calprotectin, a marker of neutrophil-driven inflammation, has shown stronger associations with acute coronary syndromes, whereas zonulin primarily reflects intestinal permeability [189]. Notably, the clinical utility of zonulin is limited by variability in assay reliability and a lack of standardized measurement protocols [138].

In the context of this review, elevated zonulin levels and, consequently, increased gastrointestinal permeability are associated with various cardiovascular diseases. It has been reported that patients with inflammatory bowel disease exhibit higher zonulin levels, suggesting that zonulin could serve as a biomarker of cardiovascular risk in these patients.

In patients with heart failure, Ahmad et al. identified a significant correlation between an increased left ventricular end-systolic dimension and a reduced left ventricular ejection fraction in individuals with elevated serum zonulin levels. These findings suggest that zonulin may play a significant role in the progression of heart failure. Additionally, it should be noted that heart failure itself can impact gut permeability, leading to intestinal edema and hypoperfusion [190].

A study found that patients with coronary artery disease exhibited elevated plasma levels of zonulin compared to controls. The authors hypothesized that increased intestinal permeability facilitates bacterial translocation, contributing to the development of atherosclerosis. This aligns with data indicating the presence of bacterial infiltration in atherosclerotic plaques, primarily originating from the gut and oral cavity [191].

Hypertension is a well-established risk factor for both atherosclerosis and heart failure. Emerging evidence suggests that hypertensive patients exhibit elevated zonulin levels, supporting the role of intestinal barrier dysfunction in the pathophysiology of cardiovascular diseases [192,193].

Most studies indicate that zonulin levels are higher in patients with IBD compared to healthy controls, suggesting its potential role as a marker of cardiovascular disease risk [194]. However, some studies have not confirmed this difference, with a possible explanation being the predominance of patients in remission [195]. Furthermore, data on the differences in zonulin levels between CD and UC remain inconsistent, highlighting the need for further research to clarify its specific role in the pathogenesis of different IBD subtypes.

Emerging biomarkers such as fecal calprotectin, short-chain fatty acids (SCFAs), and zonulin have been widely studied for their diagnostic and prognostic value in IBD. They are increasingly being investigated for their role in cardiovascular risk assessment. Importantly, these biomarkers are generally considered safe for clinical use, as their measurement relies on non-invasive sampling (e.g., stool or blood) and does not involve the administration of exogenous substances. Fecal calprotectin, for example, is a well-established, non-cytotoxic marker routinely used in clinical practice to monitor intestinal inflammation, with no reported adverse effects associated with its measurement [195]. Similarly, SCFAs are endogenous microbial metabolites, and their quantification in stool or serum poses no safety risk to patients [145]. Zonulin, a marker of intestinal permeability, is also assessed using blood or stool samples and has not been associated with cytotoxicity or adverse clinical outcomes [147]. While these biomarkers are safe and non-invasive, further studies are needed to validate their predictive value for cardiovascular risk in IBD populations and to establish standardized reference ranges for clinical use. Current evidence supports their potential utility for monitoring systemic inflammation and vascular risk. Still, large-scale prospective studies are warranted to confirm their prognostic significance and optimize their integration into routine care. Table 3. provides a comparative overview of the sensitivity, specificity, and predictive value of these biomarkers, highlighting their clinical utility.

Together, these findings suggest that gut dysbiosis in IBD not only alters the production of key microbial metabolites and host-derived biomarkers but also amplifies systemic inflammation and thrombogenic risk, ultimately converging on pathways that accelerate cardiovascular disease.

## 4. Conclusions and Future Directions

In conclusion, patients with inflammatory bowel disease (IBD) represent a unique population at significantly increased risk for the development of cardiovascular disease (CVD), a risk that extends beyond the influence of traditional cardiovascular risk factors. The pathophysiological connection between IBD and CVD is multifactorial and complex, involving chronic systemic inflammation, hypercoagulability, endothelial dysfunction, and increasingly recognized alterations in the gut microbiome. Recent evidence highlights the pivotal role of gut dysbiosis, characterized by reduced microbial diversity, shifts in dominant bacterial phyla, and impaired production of beneficial metabolites, such as short-chain fatty acids (SCFAs), in promoting increased intestinal permeability and facilitating the translocation of microbial products, including lipopolysaccharide (LPS), into the systemic circulation. This, in turn, amplifies systemic inflammation and contributes to vascular injury and atherogenesis. Although robust clinical data on specific microbial alterations and validated biomarkers remain limited, current findings suggest a meaningful correlation between IBD activity, cardiovascular risk, and emerging markers, including zonulin, SCFAs, calprotectin, and C-reactive protein (CRP).

Furthermore, the impact of IBD therapies, including corticosteroids and biologic agents, on cardiovascular health warrants careful consideration, as some treatments may exacerbate or mitigate CVD risk through their effects on inflammation and metabolic profiles. Given these complexities, there is a pressing need for future research to further elucidate the dynamic interplay between gut microbiota composition, host immune responses, and biomarker profiles in the context of cardiovascular risk among IBD patients. Such efforts will be essential not only for identifying reliable biomarkers and novel therapeutic targets but also for developing effective strategies for early intervention, risk stratification, and, ultimately, reducing cardiovascular morbidity and mortality in this growing patient population. Future research should prioritize longitudinal studies to clarify the temporal relationship between gut microbiota alterations, emerging biomarkers, and cardiovascular outcomes in patients with IBD. Large-scale, multi-omics approaches integrating microbiome, metabolome, and host inflammatory profiles are needed to identify robust, predictive biomarkers for cardiovascular risk stratification. Randomized controlled trials of microbiota-targeted interventions, such as prebiotics, probiotics, and fecal microbiota transplantation, should be conducted to evaluate their efficacy in modulating both intestinal and cardiovascular health. Additionally, efforts to standardize assays for biomarkers like zonulin will be essential for their reliable use in clinical practice. Ultimately, the development of integrated, personalized risk models that combine clinical, microbial, and biomarker data may facilitate the earlier identification and targeted prevention of cardiovascular complications in IBD.

## Figures and Tables

**Table 1:** Pathophysiological mechanisms linking inflammatory bowel disease to cardiovascular disease.

Pathway	Mechanism	Key Biomarkers/Elements	Cardiovascular Consequence	Refs.
Hypercoagulability	↑ Coagulation factors ↓ natural anticoagulants, hypofibrinolysis	Fibrinogen, Factors V/VIII/IX, Antithrombin III, PAI-1, D-dimers	Venous/arterial thrombosis ↑ VTE risk	[104,105]
Systemic Inflammation	Chronic inflammation ↑ proinflammatory cytokines	IL-6, TNF-α, CRP, ROS	Atherosclerosis, plaque instability ↑ level of tubules in stages VII/VIII	[105,106]
Endothelial Dysfunction	Impaired NO production ↑ Arginase ↓ eNOS activity	L-arginine, Arginase, NO	Early atherosclerosis, vascular tone dysregulation	[107,108,109]
Gut Barrier Dysfunction	Increased intestinal permeability due to dysbiosis	Zonulin	Endotoxemia, systemic inflammation	[110,111]
Dysbiosis	↓ SCFA-producing bacteria ↑ proinflammatory taxa	SCFAs, Firmicutes, Proteobacteria	Hypertension, arterial stiffness, metabolic dysregulation	[112,113]
Inflammatory Mediators	Neutrophil activation, activation of NF-κB, and MAPK	Calprotectin, RAGE, Toll-like receptor 4 (TLR4)	Cardiac remodeling, ↑ CVD risk	[114,115,116]

**Table 2:** Emerging biomarkers linking IBD and CVD.

Biomarker	Source	Main Producers/Origin	Role in Pathophysiology	Ref.
Short-chain fatty acids (SCFAs)	Gut microbiota (mainly *Firmicutes: Ruminococcus*, *Akkermansia*, *Bifidobacterium*, *Lactobacillus*, *Succinivibrio*, *Roseburia*, *Clostridium*, *Eubacterium*)	Produced by bacterial fermentation of dietary fibers and resistant carbohydrates in the colon (especially cecum)	Maintain the barrier, modulate inflammation, energy source for colonocytes, modulate immune homeostasis, reduce inflammation, regulate motility, promote mucus production, reduce colon cancer risk	[144]
Calprotectin	Neutrophils	Released during inflammation	Marker of intestinal inflammation, modulates leukocyte migration, cytokine expression, cell proliferation; acts intra- and extracellularly	[145]
Zonulin	Intestinal epithelium, liver	Pre-haptoglobin 2; upregulated by gliadin and bacteria via the CXCR3 receptor	Regulates gut permeability by modulating tight junctions; increased levels indicate barrier dysfunction	[146]

**Table 3:** Summary of the sensitivity, specificity, and predictive value of the most studied biomarkers in IBD and their association with cardiovascular risk.

Biomarker	Sensitivity (%)	Specificity (%)	Predictive Value for CVD in IBD	Ref.
Short-chain fatty acids (SCFAs)	80–95	70–85	High for intestinal inflammation; indirect for CVD	[193]
Fecal Calprotectin	Not Available (N/A)	N/A	High for intestinal inflammation; indirect for CVD	[194]
Zonulin	65–80	60–75	Under investigation; linked to gut permeability and CVD	[195]

## Data Availability

Not applicable.
